# Cardioembolic ST-Elevation Myocardial Infarction in a Patient With Atrial Fibrillation and Polycythemia Vera

**DOI:** 10.7759/cureus.104668

**Published:** 2026-03-04

**Authors:** Sankalp Acharya, Vyoma Patel, Sai Shahane, Dhruvil Patel, Rutuja Challawar, Hardikkumar Bhanderi

**Affiliations:** 1 Internal Medicine, Monmouth Medical Center, Long Branch, USA; 2 Internal Medicine, Maimonides Medical Center, New York, USA; 3 Internal Medicine, GMERS (Gujarat Medical Education and Research Society) Medical College, Gandhinagar, IND

**Keywords:** anticoagulation, atrial fibrillation, coronary embolism, polycythemia vera, st-elevation myocardial infarction

## Abstract

ST-elevation myocardial infarction (STEMI) is most commonly caused by acute atherosclerotic plaque rupture with superimposed thrombosis. Coronary embolism represents a rare but clinically important non-atherosclerotic mechanism of STEMI, which is frequently associated with atrial fibrillation and other prothrombotic conditions. Embolic STEMI has been associated with higher rates of adverse outcomes and requires a management strategy distinct from atherosclerotic disease.

We present a 61-year-old male with paroxysmal atrial fibrillation and polycythemia vera who presented with anterolateral STEMI. Coronary angiography revealed occlusion of the distal left anterior descending and diagonal branches without underlying atherosclerosis, consistent with a cardioembolic etiology. He was managed with anticoagulation without stenting and discharged on long-term oral anticoagulation.

This case highlights the importance of considering coronary embolism in patients with arrhythmias or hypercoagulable states presenting with STEMI. Early recognition allows avoidance of unnecessary coronary stenting, guides appropriate anticoagulation, and supports targeted strategies to prevent recurrent thromboembolic events.

## Introduction

ST-elevation myocardial infarction (STEMI) is classically attributed to acute atherosclerotic plaque rupture followed by thrombotic occlusion of a coronary artery. However, non-atherosclerotic mechanisms, including coronary embolism, spontaneous coronary artery dissection, and coronary vasospasm, account for a small but clinically significant subset of cases. Coronary embolism is an uncommon cause of STEMI. Still, it is clinically substantial, accounting for approximately 2.9% to 5% cases of acute myocardial infarction [1,2]. However, this incidence is likely underestimated. Embolic STEMI is associated with worse outcomes than atherosclerotic STEMI [2]. Management strategies differ from those for atherosclerotic STEMI, underscoring the importance of prompt recognition and appropriate management. Here, we present a case of embolic STEMI in a patient with atrial fibrillation and polycythemia vera.

## Case presentation

A 61-year-old male with a past medical history significant for paroxysmal atrial fibrillation (CHA₂DS₂-VASc score of 0), hypothyroidism, obstructive sleep apnea (OSA), and polycythemia vera presented to the emergency department with two hours of substernal chest pain radiating to the left arm. The chest pain was associated with intermittent palpitations but did not have any syncope, dyspnea, or diaphoresis. He reported his last episode of atrial fibrillation occurred three months prior, and he was not on chronic anticoagulation at the time of admission.

On arrival, the patient was hemodynamically stable, with no evidence of cardiogenic shock or acute heart failure. Telemetry showed a sinus rhythm while he was in the emergency department. Initial laboratory evaluation demonstrated significantly elevated and uptrending cardiac troponin I levels, rising from 47.8 ng/ml (0-0.04 ng/ml) to 53.7 ng/ml (0-0.04 ng/ml) and subsequently to 56.1 ng/ml (0-0.04 ng/ml). Complete blood count was notable for erythrocytosis with a hemoglobin of 18.6 g/dL (13.5-17.5 g/dL) and hematocrit of 55.5% (41-50%), consistent with his known history of polycythemia vera. The remainder of the complete blood count and comprehensive metabolic panel were within normal limits.

**Figure 1:**
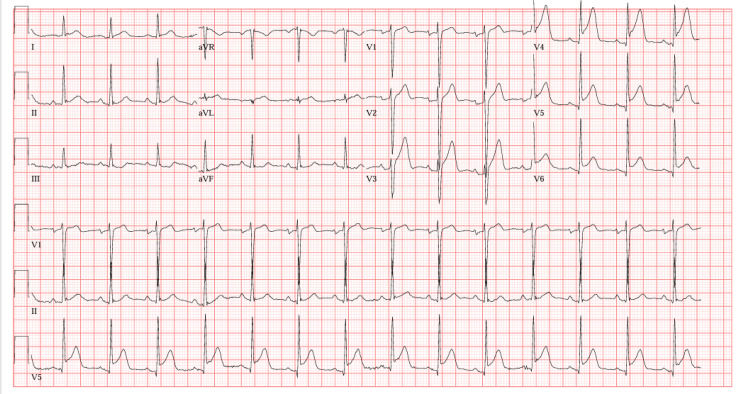
The EKG showed ST elevations in V4-V6, with reciprocal ST depression in leads II, III, and aVF.

The EKG showed ST elevations in V4-V6, with reciprocal ST depression in leads II, III, and aVF, as shown in Figure 1.

**Figure 2:**
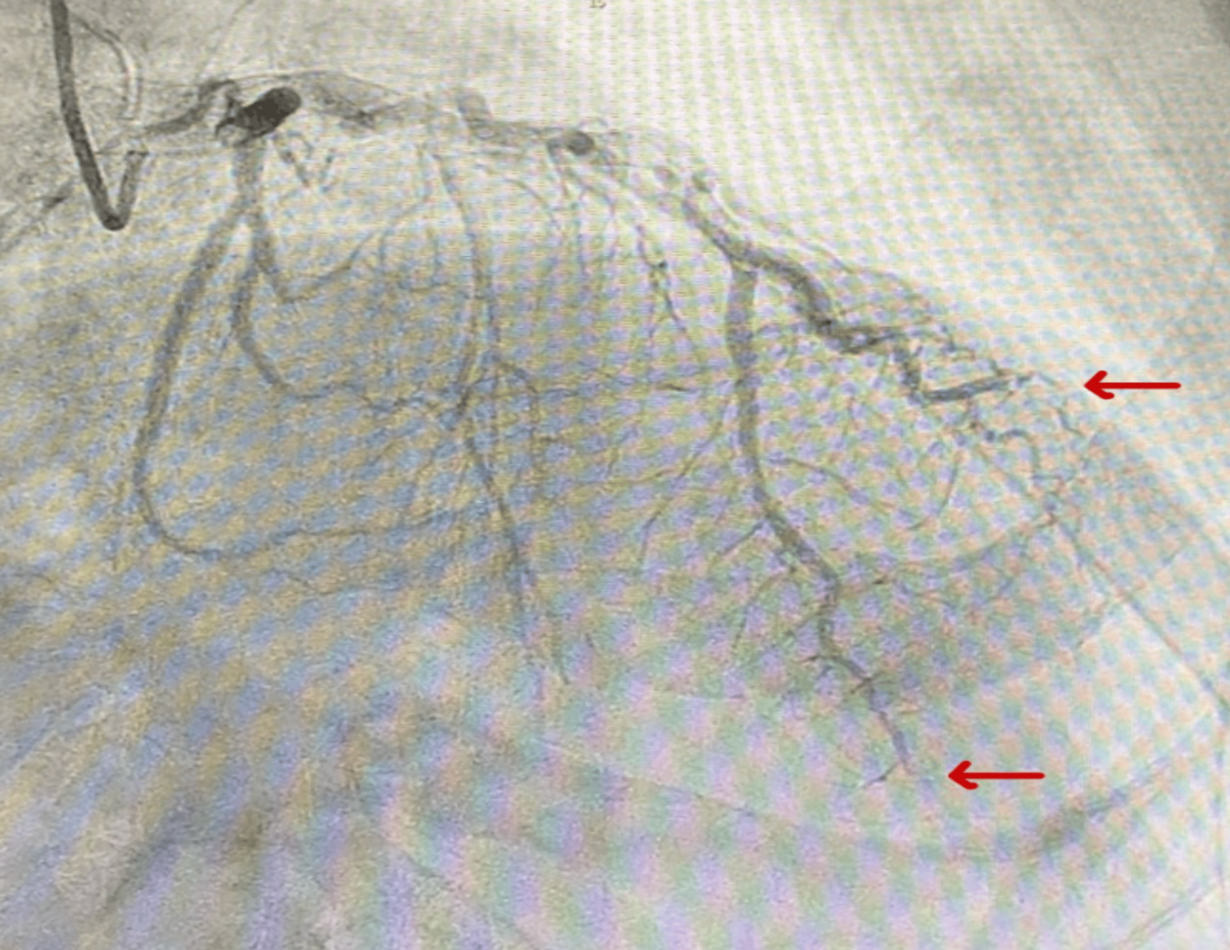
Coronary angiography revealed distal left anterior descending (LAD) and diagonal branch occlusion without plaque, suggestive of a cardioembolic etiology.

The cardiac response team was activated. He received aspirin and ticagrelor and underwent urgent coronary angiography, which revealed distal left anterior descending (LAD) and 2nd diagonal branch occlusion without plaque with TIMI (Thrombolysis in Myocardial Infarction) 0-1 flow and small thrombus burden, as shown in Figure 2, suspicious of a cardioembolic etiology. Intracoronary imaging with intravascular ultrasound (IVUS)/optical coherence tomography (OCT) was not performed. Thrombectomy was considered but not performed, as distal vessel location and small thrombus burden favored conservative anticoagulation.

No stents were placed. Further workup, including transthoracic echocardiography, showed an ejection fraction of 55-60% with apical dyskinesia, trace mitral regurgitation, mild tricuspid regurgitation, and normal right ventricular systolic pressure. Heparin infusion was initiated, and the patient was discharged on long-term anticoagulation with apixaban 5 mg twice daily. Dual antiplatelet therapy (aspirin plus ticagrelor) was discontinued after confirming embolic etiology to minimize bleeding risk, given the absence of stenting. Short-term follow-up at four weeks showed no recurrent chest pain, arrhythmia, or thromboembolic events.

## Discussion

Coronary embolism is an uncommon cause of STEMI but is clinically significant, accounting for approximately 2.9% to 5% cases of acute myocardial infarction, though this incidence is likely underestimated [1,2]. This case illustrates the importance of recognizing embolic STEMI in patients with prothrombotic conditions, as early diagnosis fundamentally alters both acute management and long-term therapeutic strategies.

Atrial fibrillation is the most common cause of coronary embolism, accounting for 73% of cases in the largest published series, followed by cardiomyopathy (9.4% to 25%) and malignancy (9.6% to 15.1%) [1]. Our patient had atrial fibrillation as well as polycythemia vera; these dual risk factors create a particularly high-risk scenario for embolic events. Polycythemia vera is a JAK2-mutated myeloproliferative neoplasm characterized by erythrocytosis and a substantially increased risk of thrombotic complications [3]. Arterial thrombosis occurs in 16% of patients with polycythemia vera, with acute coronary syndrome occurring in 8.3% of cases [3]. The thrombotic risk in polycythemia vera is multifactorial, related to increased blood viscosity, elevated hematocrit, leukocytosis, and JAK2V617F-mediated platelet activation [4]. Importantly, approximately 26.3% of patients with coronary embolism have no identifiable cause, underscoring the need for comprehensive evaluation [1].

The diagnosis of coronary embolism is challenging and requires a high index of suspicion based on clinical context and angiographic findings. The Shibata criteria provide a systematic diagnostic framework incorporating major criteria (angiographic evidence of thrombus or thrombus without significant atherosclerosis, multiple coronary territories involved, concomitant systemic embolization, histologic evidence, or identified embolic source) and minor criteria (minimal stenosis in non-culprit vessels, atrial fibrillation, or embolic risk factors) [1,2,5]. Patients meeting two or more major criteria, one major and two minor, or three minor criteria are classified as definite coronary embolism [5]. Based on one major and two minor criteria, this case fulfills the definition of definite coronary embolism according to Shibata et al. [2]. The patient met these criteria based on angiographic evidence of coronary occlusion without underlying plaque, the presence of atrial fibrillation, and polycythemia vera as a hypercoagulable state. The angiographic hallmarks that distinguish embolic from atherosclerotic STEMI include globular filling defects, saddle thrombi, or multiple filling defects in the absence of coronary atherosclerosis [1]. The distal location of occlusion, as in our patient, is characteristic of embolic events, as emboli typically lodge distally in previously normal coronary arteries and most often cause small but transmural myocardial infarctions [6]. Additionally, when a thrombectomy is performed, histologic analysis of the retrieved thrombus can provide confirmatory evidence of an embolic etiology [1]. In our case, transesophageal echocardiography was not performed, which limits definitive identification of an intracardiac thrombus source.

The management of embolic STEMI differs fundamentally from that of atherosclerotic STEMI. According to the 2024 SCAI Expert Consensus Statement, management depends on the size of the embolus, vessel flow, and myocardium at risk [7]. Distal coronary embolism or small branch vessel involvement may be conservatively managed with intravenous antithrombotic therapies, with a focus on identifying and treating the underlying thrombus source [7]. When percutaneous coronary intervention is indicated for a larger proximal thrombus burden, wire manipulation alone may open the occlusion and allow intrinsic fibrinolysis, or clot extraction devices may be needed [7].

The decision to avoid stent placement in our patient was appropriate and evidence-based. While percutaneous coronary intervention with stenting is standard in plaque-mediated infarction, embolic STEMI may be managed with thrombectomy or anticoagulation alone, particularly when the embolus is distal and there is no underlying coronary disease. Avoiding unnecessary stent placement in embolic STEMI reduces the risk of bleeding associated with dual antiplatelet therapy and allows focus on long-term anticoagulation to prevent recurrence [7,8]. Long-term anticoagulation is the cornerstone of secondary prevention in embolic STEMI. For a patient with atrial fibrillation, oral anticoagulation should be continued indefinitely to prevent recurrent thromboembolism [8]. The workup for embolic STEMI should include transthoracic echocardiography to assess for left ventricular thrombus and left atrial appendage thrombus, though transesophageal echocardiography provides superior visualization of the left atrial appendage and should be considered to evaluate for intracardiac shunt [7,8]. An extended cardiac monitor for paroxysmal atrial fibrillation and hematologic evaluation for hypercoagulable state are also recommended [7].

Embolic STEMI is associated with worse outcomes compared to atherosclerotic STEMI. In the propensity-matched cohorts, patients with coronary embolism had a significantly higher incidence of cardiac death (hazard ratio = 9.29, 95% CI = 1.13-76.5) compared to those without coronary embolism [2]. The five-year rate of major adverse cardiac and cerebrovascular events is 27.1%, with recurrent thromboembolism occurring in approximately 10% of patients on long-term follow-up [2]. The cardiac mortality rate in the coronary embolism cohort is 50% at 10 years, significantly higher than that of the general acute coronary syndrome population [8]. These poor outcomes underscore the critical importance of early recognition, targeted therapy with anticoagulation, and close long-term follow-up.

## Conclusions

This case highlights the importance of maintaining a high index of suspicion for coronary embolism in patients presenting with STEMI in the absence of clear angiographic evidence of plaque rupture, particularly in those with arrhythmias or hypercoagulable states. Application of structured diagnostic frameworks such as the Shibata criteria, consideration of intracoronary imaging to exclude occult atherosclerosis, and targeted evaluation with transesophageal echocardiography and hypercoagulable workup are essential to establish the diagnosis.

Management decisions in embolic STEMI should be individualized and are currently guided primarily by observational data and expert consensus. Avoiding unnecessary stent placement and tailoring antithrombotic therapy to balance thrombotic and bleeding risk are key principles. In our patient, anticoagulation-focused therapy was selected based on the embolic mechanism and underlying atrial fibrillation, with stable short-term follow-up. Further prospective studies are needed to develop standardized diagnostic algorithms and evidence-based antithrombotic strategies for embolic STEMI to better inform clinical practice and future guideline development.
